# Amyloidosis cutis dyschromica

**DOI:** 10.1186/1750-1172-7-95

**Published:** 2012-12-12

**Authors:** Jianjun Qiao, Hong Fang, Hongtian Yao

**Affiliations:** 1 Department of Dermatology, The First Affiliated Hospital, College of Medicine, Zhejiang University, No. 79, Qingchun Road, Hangzhou, 310003, Zhejiang Province, China; 2 Department of Pathology, The First Affiliated Hospital, College of Medicine, Zhejiang University, No. 79, Qingchun Road, Hangzhou, 310003, Zhejiang Province, China

**Keywords:** Amyloidosis cutis dyschromica, Amyloid, Pigmentation disorder, Cytokeratin, Congo red, Hereditary disease

## Abstract

**Background:**

Amyloidosis cutis dyschromica is a rarely documented variant of cutaneous amyloidosis. To date, only 26 cases have been reported.

**Objective:**

The purpose of this study was to improve the clinical and histopathological data for this variant of amyloidosis and to highlight the immunohistochemical features of the disease. The published cases were also reviewed.

**Methods:**

We performed a retrospective review of patients with amyloidosis cutis dyschromica in a single centre. The clinical, histopathological and immunohistochemical features were documented and analysed.

**Observations:**

We described 10 cases of amyloidosis cutis dyschromica. Six of them were female. Five patients were from the same family, and the other 5 were sporadic. The distinguishing features of the clinical presentation included generalised mottled hyper- and hypopigmented macules, which were asymptomatic or mild pruritic. The typical onset of the lesions occurred in childhood (n = 7) and occasionally after puberty (n = 3). No evidence of systemic amyloidosis deposition was observed in these cases of amyloidosis cutis dyschromica. Amyloid deposits were observed in the papillary dermis and were positive for the Congo red stain. An immunohistochemical study showed that the amyloid expresses cytokeratins CK34βE12 and CK5/6.

**Conclusions:**

We described the largest series of amyloidosis cutis dyschromica to date and reviewed the published patients. This rare disease is featured by generalised mottled hyper- and hypopigmented lesions, and it is a rare variant of primary cutaneous amyloidosis without evidence of systemic amyloid deposition. Positive staining for the cytokeratins CK34βE12 and CK5/6 in amyloidosis cutis dyschromica suggests that the amyloid is derived from keratinocytes.

## Background

Primary cutaneous amyloidosis refers to the extracellular deposition of amyloid material in previously apparent normal skin without systemic involvement. The major variants of primary cutaneous amyloidosis are macular and lichen [1]. Amyloidosis cutis dyschromica (ACD), first described in 1970, is a rarely documented variant of cutaneous amyloidosis [2]. Morishima described its features as the following: (i) dotted, reticular hyperpigmentation with hypopigmented macules distributed over nearly all of the body, (ii) no or little itch, (iii) onset before puberty and (iv) focal amyloid deposition under the epidermis [2]. To date, there are only 26 published cases worldwide. Some recently published ACD cases presented with new characteristics. However, the origin of the amyloid was not described in most of these published cases.

To further understand this disease, we report 10 new cases of ACD and investigate the origin of the amyloid materials. We also review the cases of ACD reported in the literature.

## Patients and methods

We performed a retrospective case analysis of 10 patients with ACD who were referred to our dermatology department from 2004 to 2012. We retrieved the patients’ clinical histories, clinical findings, laboratory results and attending physician names. All skin biopsy samples were fixed in 10% formalin and embedded in paraffin. Four- μm-thick sections were stained with hematoxylin-eosin and Congo red. A panel of cytokeratin stains (Additional file 1: Table S1), including AE1/AE3, CK5/6 and CK34βE12, was performed on the available tissues. PubMed and Google Scholar were searched for published cases in English. The study was approved by the Ethical Committee of The First Affiliated Hospital, College of Medicine, Zhejiang University. All patients gave their written informed consent.

## Results

### Demographic features

A total of 10 patients with ACD were identified in our database search from 2004 to 2012 (Additional file 2: Table S2). Four patients were male, and 6 were female. The patient age ranged from 17 to 73 years, with an average age of 38.7 years.

### Clinical presentation

The clinical features of the 10 patients are presented in Additional file 2: Table S2. Patients no. 1a to no. 1e were from the same family. There were no family members with similar disease for the other 5 patients. All patients were born to non-consanguineous parents. They had no photosensitivity, blistering eruption or short stature and reported no history of extensive sun exposure, any other cutaneous disorder or systemic disease before the onset of the lesions.

**Figure 1:**
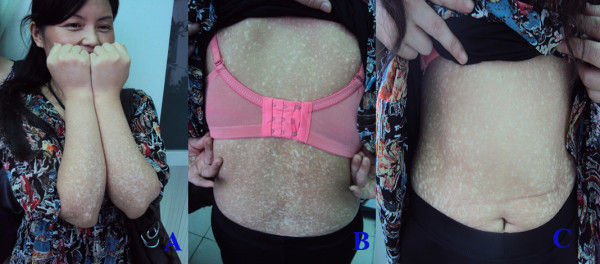
**Clinical presentation of amyloidosis cutis dyschromica. A** and **B**, Generalised mottled hyper- and hypopigmented macules were distributed over the entire body (patient 1b). **C**, However, the lesions on the face and distal extremities were mild.

**Figure 2:**
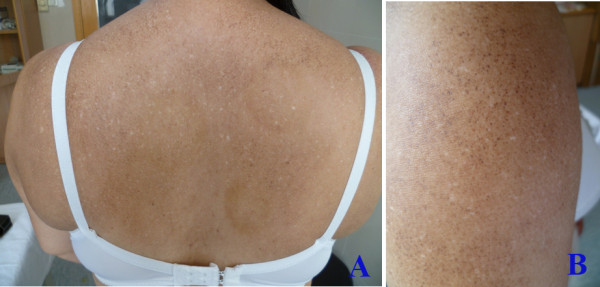
**Clinical presentation of amyloidosis cutis dyschromica.** In addition to the hyper- and hypopigmented macules, diffuse hyperpigmented small papules were noted (patient 4).

The cutaneous lesions were asymptomatic or mild pruritic. The typical lesion onset occurred in childhood (n = 7) and occasionally after puberty (n = 3). The eruptions progressed gradually over years to involve almost the entire body and stabilised thereafter. The time elapsed from the initial presentation to the final diagnosis varied from 4 years to more than 50 years. The distinguishing features of the clinical presentation included generalised mottled hyper- and hypopigmented macules (Figure 1A, 1B and 1C). Diffuse hyperpigmented small papules were noted in one patient (Figure 2A, 2B). The size of the macules and papules ranged from 2 to 10 mm, and they involved almost the entire body in a symmetrical pattern. The lesions were more pronounced on the trunk and the limbs with relative sparing of the face, hands, feet and neck in all cases (Figure 1C). There was no telangiectasia, erythema, or atrophy. The nails, hair, teeth, mucous membranes, soles and palms had a normal appearance. The systematic examination was unremarkable except for one patient with colon cancer.

Laboratory investigations, including blood cell count, urine analysis, abdominal ultrasonography, and serum protein electrophoresis, revealed no abnormality.

### Histopathological findings

**Figure 3:**
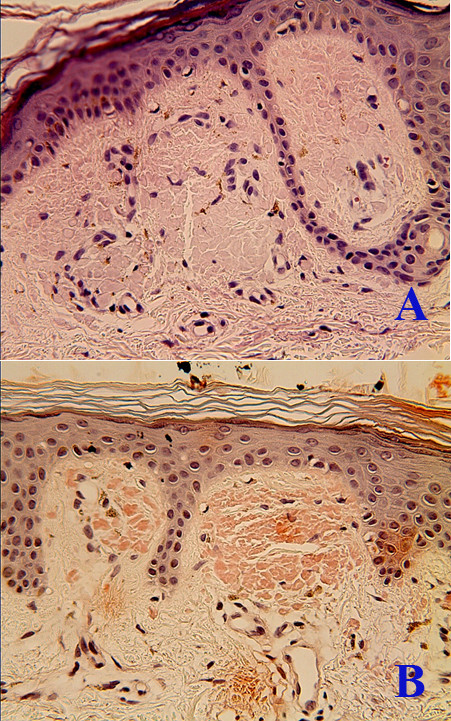
**Histopathological manifestation of amyloidosis cutis dyschromica. A**, Histopathology of amyloidosis cutis dyschromica skin lesions showed homogenous eosinophilic masses in the papillary dermis and sparse melanophages in the superficial dermis (patient 1b; hematoxylin-eosin, original magnification: ×400). **B**, The Congo red stain showed positivity in the papillary dermal deposits (original magnification × 400 magnification).

**Figure 4:**
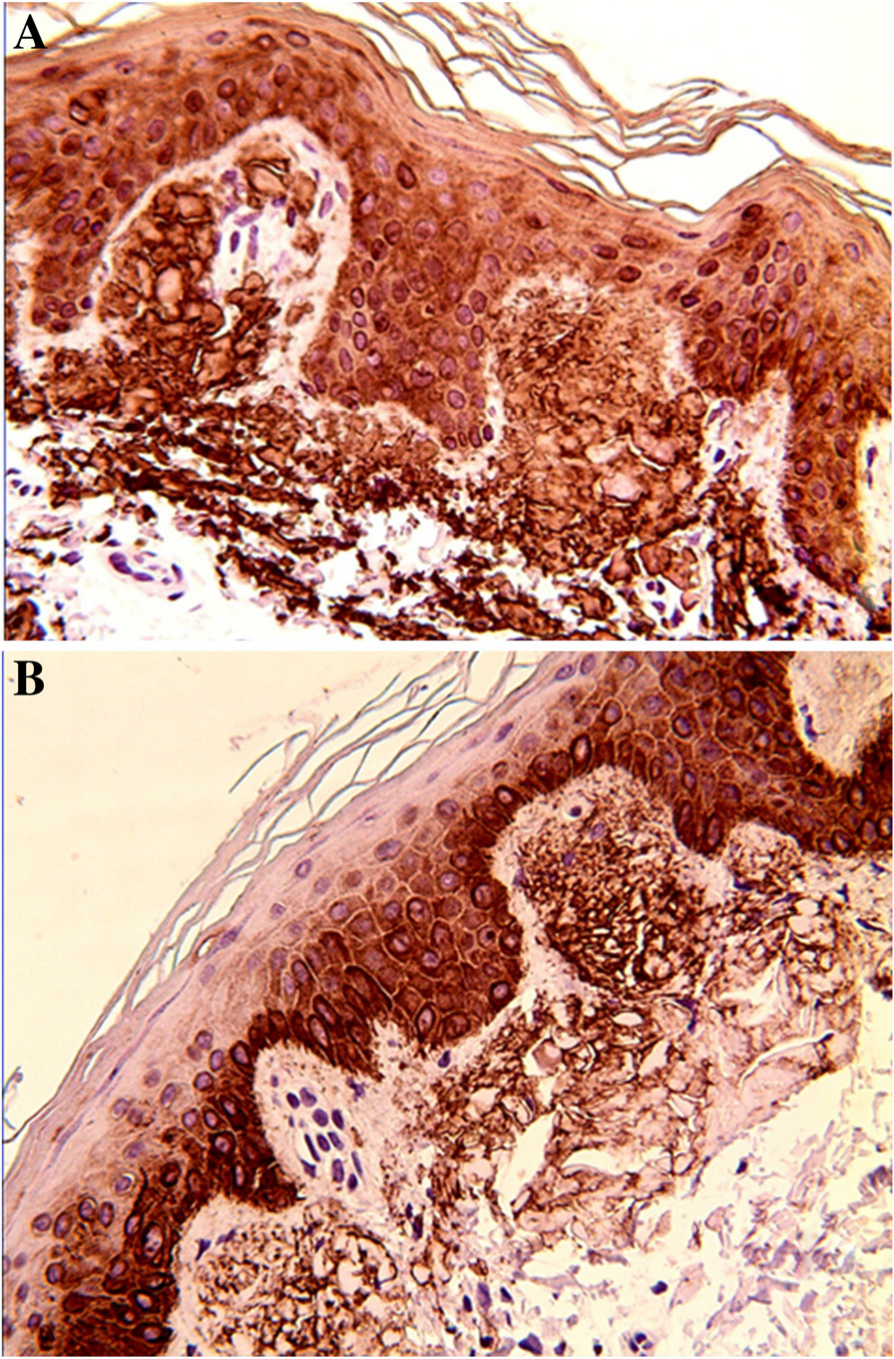
**Immunohistochemical profile of amyloidosis cutis dyschromica.** Cytokeratin staining showed that the papillary dermal deposits were strongly positive for CK34βE12 and CK5/6 in amyloidosis cutis dyschromica. (patient 5; **A**, CK34βE12 stain; **B**, CK5/6 stain; original magnifications: **A**, × 400, **B**, × 400).

The histopathological findings of specimens from hyper- and hypopigmented lesions in all cases indicated homogenous eosinophilic masses in the papillary dermis and sparse melanophages in the superficial dermis (Figure 3A). In addition to the features observed in the hyper- and hypopigmented lesions, specimens from the lichenoid papules showed mild hyperkeratosis. There were no significant alterations in the reticular dermis. The eosinophilic material stained positive for Congo red in all cases (Figure 3B). Similar cytokeratin staining profiles were found in all cases, with strong positive staining for CK34βE12 and CK5/6 and faint positivity or absence of staining for AE1/AE3 (Figure 4A, B).

### Treatment and outcome

Oral vitamin E and vitamin C were the treatment of choice in most cases and resulted in minimal improvement. Three patients received oral acitretin at 20 mg per day; two had a good response within three months, but the other demonstrated minimal improvement.

### Literature findings

**Figure 5:**
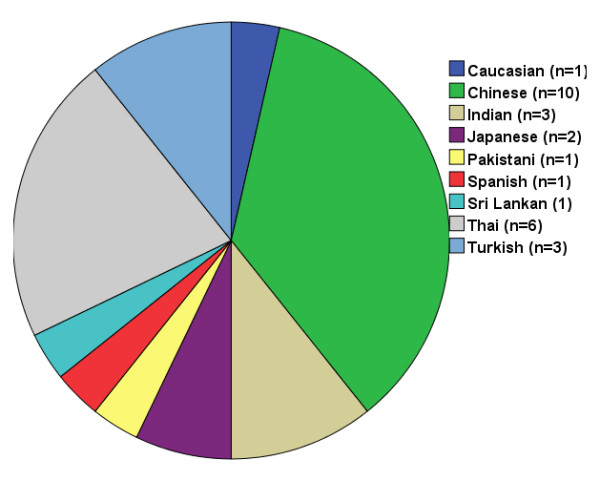
**Ethnic distribution of 26 cases of amyloidosis cutis dyschromica from the literature.** Most published cases of amyloidosis cutis dyschromica observed in Asian patients.

The literature data are summarised in Additional file 3: Table S3. A total of 26 published cases were identified in a literature search [2-17]. The ethnic distribution of the 26 cases is shown in Figure 5. Four patients did not have family members with the disease, and the other 21 cases were familial and distributed among 12 families.

## Discussion

We report the largest series of ACD cases to date. Similar to macular amyloidosis and lichen amyloidosis, the Asian population is more susceptible to this disease, particularly Chinese, Japanese and Thai individuals. ACD was familial in 19 of 36 patients (10 in this series and 26 in the literature); 17 cases were sporadic. However, only two siblings had consanguineous parents. The onset of dyschromia occurred before puberty in most patients and after puberty in only 5 patients (3 patients in our series and 2 patients in the literature).

Itch is a significant symptom in patients with macular amyloidosis and lichen amyloidosis, which are the two most common variants of primary cutaneous amyloidosis [18]. The lesions of ACD are generally asymptomatic, but patients may express cosmetic concerns. Only 5 patients had a mild itch (2 in our series, 3 in the literature).

In addition to the characteristic generalised reticular hyperpigmentation checked with hypopigmented macules, small hyperpigmented papules were found in 5 patients (1 in our series, 5 in the literature). A clinical morphology overlap between ACD and other variants of primary cutaneous amyloidosis has been observed in published cases [3,6,13]. Presentations of blister (n = 2) [3], freckles (n = 1) [13] and telangiectasia (n = 2) [13] have been described. Concurrent with ACD with lichen, poikiloderma-like, dyschromic and bullous variant of primary cutaneous amyloidosis was reported in one patient [6]. Our series lacked any concurrent mucocutaneous disorders, and the hair, nails, teeth, membranes, palms, and soles were normal in all of our patients. Erythema was not found in any of the ACD patients.

Although ACD is widely accepted as a primary cutaneous amyloidosis, it has been associated with other diseases in a few cases. One case of ACD with generalised morphea was reported [13]. Two siblings presented ACD associated with atypical Parkinsonism, spasticity and motor weakness [5]. Systemic amyloid deposition could potentially explain the extracutaneous manifestations of the siblings, but neurological investigations revealed no evidence of systemic amyloid deposition [5]. The authors postulate that the syndrome in this family may have been caused by a concomitant mutation of closely linked genes [5]. In our series, colon carcinoma was found in one patient when he was 16 years old. The mechanism of the association is unknown. An ACD diagnosis may cause concern for treating clinicians because of the possibility of underlying systemic amyloidosis. However, no systemic involvement was detected in any of the reported ACD patients.

The histopathological findings in our series are similar to those reported in the literature, with amyloid depositing in the papillary dermis. Amyloid was observed in the superficial dermis in 2 published cases [10,18]. It is not clear why the amyloid deposition was so extensive in the two patients. Histopathologically papular ACD lesions also revealed amyloid deposits in the papillary dermis.

The presentation of melanophages in the superficial dermis suggests damage to the basal epidermis in ACD, lichen amyloidosis and macular amyloidosis. Although reports examining the origin of ACD amyloid are rare, the keratin origin of amyloid has been demonstrated in lichen amyloidosis and macular amyloidosis [18]. The damage may then result in keratinocyte deposition and degenerate to amyloid at the dermo-epidermal junction [18]. Only 3 reports in the literature reported the keratin composition of the amyloid in ACD [5,12,16]. However, only wide-spectrum cytokeratin antibodies or high molecular weight cytokeratin antibodies were used in these reports. Wide-spectrum anti-cytokeratin antibodies were positive in two of the three cases [12,16], and high molecular weight cytokeratin was positive in another patient [5]. In our series, 100% (6/6) of the cases showed immunohistochemical positivity for CK34βE12 and CK5/6 and negative or faint positive staining for pan-cytokeratin AE1/AE3. To our knowledge, this is the first report showing the amyloid staining profile of CK34βE12 and CK5/6 in ACD. The immunohistopathological results and limited literature reports strongly support an epidermal origin of the amyloid.

The occurrence of familial cases and disease onset before puberty suggests a genetic aetiology of ACD. The genetic basis of ACD is not known. It has been suggested that genetic factors may lead to the disruption of DNA repair in keratinocytes [16]. Moriwaki et al. observed slow DNA repair in keratinocytes due to ultraviolet C damage and reduced repair following ultraviolet B damage [16]. However, the cutaneous findings of all patients are distributed over the entire skin surface, and the face, neck, and hands often have a milder presentation than the non-sun-exposed skin area. This argues against a primary role of photosensitivity in this disorder.

The differential diagnosis of dyschromatoses is often a challenge to clinical dermatologists, particularly in ethnic populations in which this disease is not commonly observed [19]. The differential diagnosis of ACD includes other diseases presenting as dyschromatosis and other amyloid deposition disorders, including dyschromatosis universalis hereditaria, xeroderma pigmentosum, and poikiloderma-like cutaneous amyloidosis [20]. Dyschromatosis universalis hereditaria is also characterised by the presence of generalised hypopigmented and hyperpigmented macules, and it begins before puberty. However, histologically, no amyloid deposition has been observed in dyschromatosis universalis hereditaria [21]. Xeroderma pigmentosum may have a similar dyschromatosis appearance but with marked photosensitivity [22]. There is also evidence of actinic damage beginning in infancy and early childhood, and skin cancers often develop during the first decade of life [22]. The clinical features of poikiloderma-like cutaneous amyloidosis are similar to those of ACD [23]. Poikiloderma-like cutaneous amyloidosis is characterised by the presence of reticular hyper- and hypopigmentation lesions, atrophy and telangiectasia. However, many of the published patients did not fully express the triad. Diffuse hyperpigmentation was reported in all described patients, with telangiectasia, atrophy and lichenoid papules in some. This variant is associated with photosensitivity, short stature and palmoplantar keratoderma [23,24]. A few clinical features overlap between poikiloderma-like cutaneous amyloidosis and ACD. For example, dotty atrophy and vesicles were observed in two siblings with ACD [3], and telangiectasia and photosensitivity were observed in one patient with ACD [16].

The treatment of ACD is not well documented. Success has been reported with photoprotection, antioxidants and acitretin in some case reports [12]. We used acitretin in three patients in our series, and a significant improvement of the skin appearance was observed in two. Therefore, acitretin may be a promising drug for treating the disease.

A major limitation of our series and the published literature regarding ACD continues to be small sample size. ACD is a rare disease; therefore, it is very difficult to identify sample sizes large enough to reach significant conclusions.

## Conclusions

We report 10 new cases of ACD. The distinguishing features of the clinical presentation included generalised mottled hyper- and hypopigmented macules. No evidence of systemic amyloidosis deposition was found in these patients. Amyloid deposits were observed in the papillary dermis. Positive staining for cytokeratins CK34βE12 and CK5/6 in amyloidosis cutis dyschromica suggests that the amyloid is derived from keratinocytes.

## Abbreviations

ACD: Amyloidosis cutis dyschromica; CK: Cytokeratin.

## Competing interests

All authors declare that they have no competing interests.

## Authors’ contributions

JQ and HF collected the clinical data. JQ drafted the manuscript. HY carried out the pathological and immunohistochemical assays. JQ, HF, and HY participated in the design of the study. JQ conceived of the study. HF and HY revised the manuscript. All authors read and approved the final manuscript.

## Supplementary Material

Additional file 1 **Table S1.** The manufacturers of the antibodies used in this study and the keratins recognised. Click here for file

Additional file 2 **Table S2.** Clinical and histological data of patients with amyloidosis cutis dyschromica: current series. Click here for file

Additional file 3 **Table S3.** Clinical and histological data of patients with amyloidosis cutis dyschromica: a review of published cases. Click here for file
