# Supplementary material for: Amyloidosis cutis dyschromica
Source: Orphanet J Rare Dis. 2012 Dec 12;7:95. doi: 10.1186/1750-1172-7-95 (PMC3554482; doi:10.1186/1750-1172-7-95)
Supplement: Additional file 1 — Table S1. The manufacturers of the antibodies used in this study and the keratins recognised. [file 1750-1172-7-95-S1.doc]

**Additional file 1**

**Table S1 The manufacturers of the antibodies used in this study and the keratins** recognised

| **Antibodies** | **Recognised cytokeratin** | **Manufacturer** |
| --- | --- | --- |
| AE1/AE3 | pan cytokeratin | Shanghai Long Island Biotec.CO.,LTD |
| CK5/6 | cytokeratins 5 and 6 | DAKO |
| CK34βE12 | cytokeratins 1, 5, 10, and 14 | DAKO |
