# Supplementary material for: Amyloidosis cutis dyschromica
Source: Orphanet J Rare Dis. 2012 Dec 12;7:95. doi: 10.1186/1750-1172-7-95 (PMC3554482; doi:10.1186/1750-1172-7-95)
Supplement: Additional file 2 — Table S2. Clinical and histological data of patients with amyloidosis cutis dyschromica: current series. [file 1750-1172-7-95-S2.doc]

**Additional file 2**

**Table S2 Clinical and histological data of patients with amyloidosis cutis dyschromica: current series**

| **Patient no./Sex/Age, year** | **Onset age, year** | **Familial** | **Consanguineous parents** | **Symptom** | **Cutaneous findings** | **Mucous membranes, nails, hair, palms, and soles** | **Systemic examination** | **Congo red** | **AE1/AE3** | **CK5/6** | **CK34ßE12** |
| --- | --- | --- | --- | --- | --- | --- | --- | --- | --- | --- | --- |
| 1a/F/41 | 20 | Yes | No | Asymptomatic | hyper- and hypopigmented macules | Normal | Normal | - | - | - | - |
| 1b/F/44 | 10 | Yes | No | Asymptomatic | hyper- and hypopigmented macules | Normal | Normal | Positive | Negative | Positive | Positive |
| 1c/F/46 | 10 | Yes | No | Asymptomatic | hyper- and hypopigmented macules | Normal | Normal | - | - | - | - |
| 1d/M/49 | teenage | Yes | No | Asymptomatic | hyper- and hypopigmented macules | Normal | Normal | - | - | - | - |
| 1e/M/73 | teenage | Yes | No | Asymptomatic | hyper- and hypopigmented macules | Normal | Normal | - | - | - | - |
| 2/F/20 | Shortly after birth | No | No | Asymptomatic | hyper- and hypopigmented macules | Normal | Normal | Positive | Negative | Positive | Positive |
| 3/M/17 | Shortly after birth | No | No | Asymptomatic | hyper- and hypopigmented macules | Normal | Colonal cancer (found in a 16 year old) | Positive | Negative | Positive | Positive |
| 4/F/46 | 35 | No | No | Asymptomatic | hyper- and hypopigmented macules, hyperpigmented lichenoid papules | Normal | Normal | Positive | Faint positive | Positive | Positive |
| 5/F/29 | 9 | No | No | Mild itch | hyper- and hypopigmented macules | Normal | Normal | Positive | Negative | Positive | Positive |
| 6/M/22 | 18 | No | No | Mild itch | hyper- and hypopigmented macules | Normal | Normal | Positive | Faint positive | Positive | Positive |

-, Not detected
