# Supplementary material for: Amyloidosis cutis dyschromica
Source: Orphanet J Rare Dis. 2012 Dec 12;7:95. doi: 10.1186/1750-1172-7-95 (PMC3554482; doi:10.1186/1750-1172-7-95)
Supplement: Additional file 3 — Table S3. Clinical and histological data of patients with amyloidosis cutis dyschromica: a review of published cases. [file 1750-1172-7-95-S3.doc]

**Additional file 3**

**Table S3 Clinical and histological data of patients with amyloidosis cutis dyschromica: a review of published cases**

| **Source** | **Case no./Sex/**  **age, year** | **Origin** | **onset age, year** | **familial** | **consanguineous parents** | **symptom** | **morphology** | **Affected location** | **Mucous membranes, nails, hair palms, and soles** | **Complications** | **Congo red** | **CK** |
| --- | --- | --- | --- | --- | --- | --- | --- | --- | --- | --- | --- | --- |
| Garg et al. | 1/F/19 | Indian | 4 | No | No | asymptomatic | hyper- and hypopigmented macules, papules | Almost the entire body, no foot or hand involvement | NR | No | - | - |
| Fernandes et al. | 2/F/40 | Pakistani | shortly after birth | Yes | - | asymptomatic | hyper- and hypopigmented macules | all over the body | - | Parkinsonism, spasticity, motor weakness | positive | Positive (high molecular weight CK) |
| Yang et al. | 4/F/34 | Chinese | 10 | Yes | No | mild pruritic | hyper- and hypopigmented macules, blister | Almost the entire body | Normal | No | positive | - |
| Yang et al. | 5/F/39 | Chinese | 10 | Yes | No | mild pruritic | hyper- and hypopigmented macules, blister | Almost the entire body, sparing the face and neck | Normal | No | positive | - |
| Chandran et al. | 6/F/27 | Chinese | 17 | No | No | - | hyper- and hypopigmented macules accompanying lichen, poikiloderma-like, and bullous variants | neck and limbs | Normal | No | positive | - |
| Madarasingha et al. | 7/M/36 | Sri Lankan | childhood | - | - | asymptomatic | hyper- and hypopigmented macules and papules | Almost the entire body, sparing the face | Normal | No | - | - |
| Karadag et al. | 6/F/16 | Turkish | - | Yes | Yes | asymptomatic | hyper- and hypopigmented macules | the entire body | Normal | No | - | - |
| Karadag et al. | 7/M/22 | Turkish | 7 | Yes | Yes | asymptomatic | hyper- and hypopigmented macules | the entire body except the face | - | No | - | - |
| Huang et al. | 8/M/25 | Chinese | 8 | Yes | No | asymptomatic | hyper- and hypopigmented macules | Almost the entire body, sparing the neck, face, hands, and feet | Normal | No | positive | - |
| Huang et al. | 9/F/26 | Chinese | 8 | Yes | No | asymptomatic | hyper- and hypopigmented macules | Almost the entire body, sparing the neck, face, hands, and feet | Normal | No | positive | - |
| Huang et al. | 10/M/20 | Chinese | 9 | Yes | No | asymptomatic | hyper- and hypopigmented macules | Almost the entire body, sparing the neck, face, hands, and feet | Normal | No | positive | - |
| Huang et al. | 11/M/14 | Chinese | 11 | Yes | No | asymptomatic | hyper- and hypopigmented macules | only the lower legs | Normal | No | positive | - |
| Ho et al. | 12/F/54 | Chinese | 34 | - | - | asymptomatic | hyper- and hypopigmented macules | face, neck, axilla, trunk and limbs | - | No | positive | - |
| Wu et al. | 12/M/25 | Chinese | 8 | Yes | No | itch | hyper- and hypopigmented macules, papules | Almost the entire body, sparing the neck, face, and feet | Normal | No | positive | - |
| Wu et al. | 13/F/26 | Chinese | 8 | Yes | No | itch | hyper- and hypopigmented macules, papules | almost the entire body | - | - | - | - |
| Ozcan et al. | 14/M/24 | Turkish | nr | No | No | asymptomatic | hyper- and hypopigmented macules | almost the entire body | Normal | No | negative | AE1/AE3 positive |
| Morales Callaghan et al. | 21/F/52 | Spanish | 20 | Yes | nr | asymptomatic | hyper- and hypopigmented macules | the entire body | Normal | generalised morphea | positive | - |
| Choonhakarn et al. | 15/M/18 | Thai | 11 | Yes | No | asymptomatic | hyper- and hypopigmented macules, papules | face, trunk, extremities | Normal | No | positive | - |
| Choonhakarn et al. | 16/F/24 | Thai | 10 | Yes | No | asymptomatic | hyper- and hypopigmented macules | abdomen, lower extremities | Normal | No | positive | - |
| Choonhakarn et al. | 17/M/37 | Thai | 7 | Yes | No | asymptomatic | hyper- and hypopigmented macules | face, trunk, extremities | Normal | No | positive | - |
| Choonhakarn et al. | 18/F/42 | Thai | 12 | Yes | No | asymptomatic | hyper- and hypopigmented macules | face, trunk, extremities | Normal | No | positive | - |
| Choonhakarn et al. | 19/M/45 | Thai | 12 | Yes | No | asymptomatic | hyper- and hypopigmented macules | abdomen, buttocks, lower extremities | Normal | No | positive | - |
| Choonhakarn et al. | 20/M/58 | Thai | 9 | Yes | No | asymptomatic | hyper- and hypopigmented macules | face, trunk, extremities | Normal | No | positive | - |
| Vjaikumar et al. | 22/M/25 | Indian | 15 | Yes | No | mild pruritic | hyper- and hypopigmented macules | all over the body | Normal | No | positive | - |
| Vjaikumar et al. | 23/M/20 | Indian | 15 | Yes | No | asymptomatic | hyper- and hypopigmented macules | Trunk, sparing face and limbs | Normal | No | - | - |
| Moriwaki et al. | 24/F/24 | Japanese | 10 | No | No | asymptomatic | hyper- and hypopigmented macules, freckles, telangiectasia | primarily on the extensor surfaces of both the upper and lower extremities | - | photosensitivity | positive (pagoda red) | positive |
| Eng et al. | 25/M/42 | Caucasian | childhood | Yes | - | asymptomatic | hyper- and hypopigmented macules | everywhere in the skin | Normal | secondary amyloidosis in fibrotic pulmonary tissue | positive | - |
| Morishima | 26/F/UN | Japanese | childhood | No | Unknown | asymptomatic | hyper- and hypopigmented macules | extremities | Normal | No | positive | - |

-: Not reported.

**SUPPLEMENTARY REFERENCES**

1. Garg T, Chander R, Jabeen M, Barara M, Mittal K, Jain M, Puri V: **Amyloidosis cutis dyschromica: a rare pigmentary disorder.** *J Cutan Pathol* 2011, **38:**823-826.

2. Fernandes NF, Mercer SE, Kleinerman R, Lebwohl MG, Phelps RG: **Amyloidosis cutis dyschromica associated with atypical Parkinsonism, spasticity and motor weakness in a Pakistani female.** *J Cutan Pathol* 2011, **38:**827-831.

3. Yang W, Lin Y, Yang J, Lin W: **Amyloidosis cutis dyschromica in two female siblings: cases report.** *BMC Dermatol* 2011, **11:**4.

4. Chandran NS, Goh BK, Lee SS, Goh CL: **Case of primary localized cutaneous amyloidosis with protean clinical manifestations: lichen, poikiloderma-like, dyschromic and bullous variants.** *J Dermatol* 2011, **38:**1066-1071.

5. Madarasingha NP, Satgurunathan K, De Silva MV: **A rare type of primary cutaneous amyloidosis: amyloidosis cutis dyschromica.** *Int J Dermatol* 2010, **49:**1416-1418.

6. KARADAĞ AS, ŞİMŞEK GG: **Familial amyloidosis cutis dyschromica.** *Turk J Med Sci* 2010, **40**.

7. Huang WH, Wu CY, Yu CP, Chiang CP: **Amyloidosis cutis dyschromica: four cases from two families.** *Int J Dermatol* 2009, **48:**518-521.

8. Ho MS, Ho J, Tan SH: **Hypopigmented macular amyloidosis with or without hyperpigmentation.** *Clin Exp Dermatol* 2009, **34:**e547-551.

9. Wu CY, Yu CP, Chiang CP: **Familial amyloidosis cutis dyschromica-a case report and review of the literature.** *Dermatol Sinica* 2008, **26:**16-21.

10. Ozcan A, Senol M, Aydin NE, Karaca S: **Amyloidosis cutis dyschromica: a case treated with acitretin.** *J Dermatol* 2005, **32:**474-477.

11. Morales Callaghan AM, Vila JB, Fraile HA, Romero AM, Garcia GM: **Amyloidosis cutis dyschromica in a patient with generalized morphoea.** *Br J Dermatol* 2004, **150:**616-617.

12. Choonhakarn C, Wittayachanyapong S: **Familial amyloidosis cutis dyschromica: six cases from three families.** *J Dermatol* 2002, **29:**439-442.

13. Vijaikumar M, Thappa DM: **Amyloidosis cutis dyschromica in two siblings.** *Clin Exp Dermatol* 2001, **26:**674-676.

14. Moriwaki S, Nishigori C, Horiguchi Y, Imamura S, Toda K, Takebe H: **Amyloidosis cutis dyschromica. DNA repair reduction in the cellular response to UV light.** *Arch Dermatol* 1992, **128:**966-970.

15. Eng AM, Cogan L, Gunnar RM, Blekys I: **Familial generalized dyschromic amyloidosis cutis.** *J Cutan Pathol* 1976, **3:**102-108.

16. Morishima T: **A clinical variety of localized cutaneous amyloidosis characterized by dyschromia (amyloidosis cutis dyschromica).** *Jpn J Dermatol Series B* 1970, **80:**43-52.
